# Cardiac resynchronization therapy by pacing the right ventricular dorsal site of inflow and anterior outflow for congenitally corrected transposition of the great arteries: a case report

**DOI:** 10.1093/ehjcr/ytae607

**Published:** 2024-11-14

**Authors:** Shigehito Baba, Aya Miyazaki, Toru Watanabe, Shuichi Shiraishi, Akihiko Saitoh

**Affiliations:** Department of Pediatrics, Niigata University, 757 Asahimachidori Ichibancho, Niigata City, Niigata 951-8510, Japan; Department of Adult Congenital Heart Disease and Department of Pediatric Cardiology, Seirei Hamamatsu General Hospital, 2-12-12 Sumiyoshi, Hamamatsu City, Shizuoka 430-8558, Japan; Department of Cardiology, Niigata Prefectural Central Hospital, 205 Shinnancho, Joetsu City, Niigata 943-0192, Japan; Department of Cardiovascular Surgery, Niigata University, 757 Asahimachidori Ichibancho, Niigata City, Niigata 951-8510, Japan; Department of Pediatrics, Niigata University, 757 Asahimachidori Ichibancho, Niigata City, Niigata 951-8510, Japan

**Keywords:** Cardiac resynchronization therapy, Congenitally corrected transposition of the great arteries, Complete atrioventricular block, Case report

## Abstract

**Background:**

Evidence regarding cardiac resynchronization therapy (CRT) for congenitally corrected transposition of the great arteries (ccTGA) is insufficient. The timing to perform CRT and optimal pacing sites have not been systematically studied. We performed CRT for ccTGA with a complete atrioventricular block (CAVB) by pacing the dorsal site of right ventricular inflow (dRVI) and anterior RV outflow tract (aRVOT).

**Case summary:**

We examined a man aged 19 with ccTGA (S.L.L) and Ebstein anomaly, who developed CAVB at 19. We decided to implant CRT rather than a conventional pacemaker for preventing right ventricular (RV) dysfunction. At first, we implanted transvenous pacing leads on the right atrium and dRVI via the coronary sinus. During dRVI pacing, the most delayed contraction site was the aRVOT by the echocardiographic speckle tracking and the electrophysiological study. Accordingly, we implanted additional epicardial lead in the aRVOT and completed the implantation of CRT. After the CRT, the QRS duration was shortened from 187 to 132 ms and RV ejection fraction (RVEF) by right ventriculography increased from 35% to 42%.

The distance between two ventricular leads (dRVI and aRVOT) was 93% with 85% of longitudinal and radial direction in the RV. The effective CRT in this case was characterized by covering RV in the longitudinal and radial direction.

**Conclusion:**

Separate two-point pacing on the dRVI and aRVOT, which assists the contraction in the longitudinal and radial dimension, is considered a potential position for CRT pacing and an effective method in ccTGA.

**Learning points**

- Pacing the dorsal site of the RV inflow and anterior RV outflow tract as far as possible in the longitudinal and radial directions assisted longitudinal and radial RV contraction and improved electrical dyssynchrony.
- Pacing at these two points may be a reproducible and effective CRT in other ccTGA cases.

## Introduction

As univentricular pacing for congenitally corrected transposition of the great arteries (ccTGA) may lead to right ventricular (RV) dysfunction, cardiac resynchronization therapy (CRT) is recommended.^1,2^ The appropriate pacing site for CRT of the RV is unknown, and CRT does not always improve RV dysfunction. We performed CRT for ccTGA with complete atrioventricular block (CAVB) by pacing the dorsal site of RV inflow (dRVI) and anterior RV outflow tract (aRVOT) and subsequently evaluated the impact of the CRT on RV contraction. These pacing sites may be beneficial for CRT in ccTGA.

## Summary figure

**Figure:**
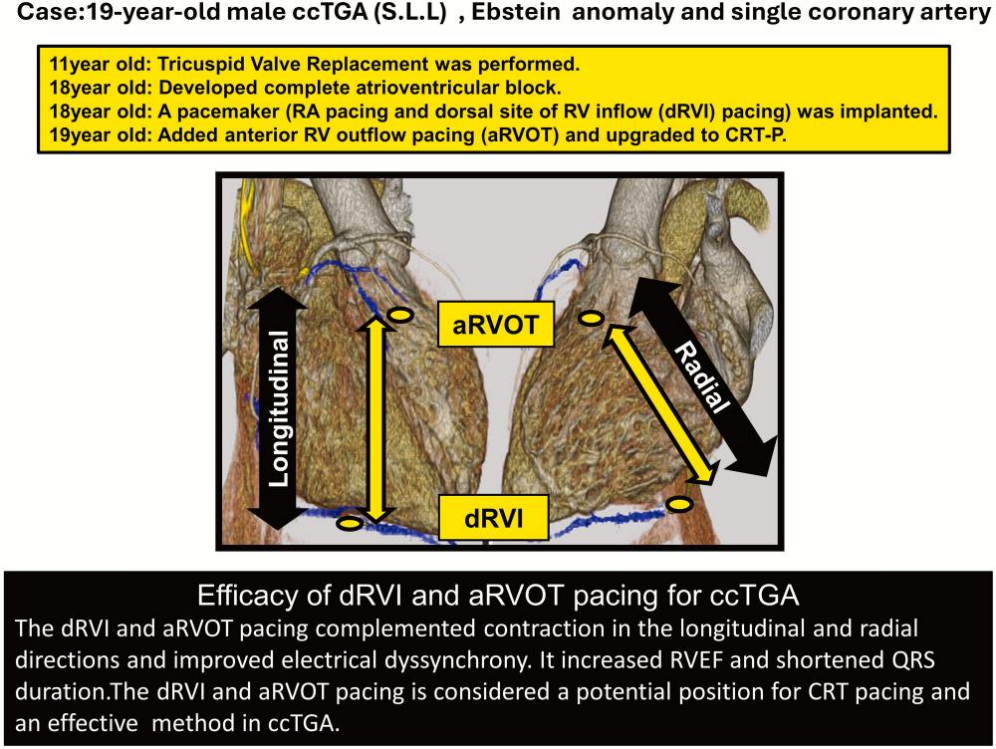


## Case presentation

A man aged 19 was diagnosed with ccTGA (S.L.L) and Ebstein anomaly, with a history of tricuspid valve replacement and RV plication at 11 years of age. In the 7 years from the surgery, the PR duration on the electrocardiogram (ECG) was approximately 180 ms and the QRS duration slowly prolonged from 110 to 136 ms with left-sided branch-bundle-block pattern. A 24-hour Holter ECG performed every 2–3 years did not detect atrioventricular (AV) block. An annual transthoracic echocardiography displaying RV ejection fraction (RVEF) ranged from 47% to 60%. The last cardiac catheterization performed when he was 15 years old revealed a RV end-diastolic volume index (RVEDVI) of 77 mL/m^2^ and RVEF of 50% by right ventriculography (RVG).

He was diagnosed with CAVB at a routine examination when he was 18 years old without a history of fatigue or fainting. During his physical examination, his heart rate was 50 b.p.m., and the heart rhythm was irregular. No increase in heart rate was observed in the exercise stress test and a 24-hour Holter ECG indicated average heart rate as 49 b.p.m., with the minimum and maximum heart rate as 37 and 62 b.p.m. Echocardiography revealed a RV diameter at end-diastole diameter (RVDd) of 42.2 mm and systolic RV diameter at end-systole (RVDs) of 28.5 mm, with an RVEF of 69.2%. Based on these findings, ventricular pacing was considered necessary; therefore, we planned pacemaker implantation. Left bundle branch area pacing or CRT was considered for ventricular pacing to prevent RV dyssynchrony. Since the long-term results for left bundle branch area pacing were still unknown, and the reports in congenital heart disease were limited, we decided to perform CRT, instead of left bundle branch area pacing. Subsequent cardiac catheterization was performed to confirm the morphology and running of the coronary veins. Coronary angiography and coronary sinus angiography indicated that the distal site of the coronary sinus and the cardiac vein at the RV-free wall were hypoplastic (see Supplementary material online, *Video S1*); thus, CRT using only transvenous leads was not feasible. Therefore, we planned to use both transvenous endocardial and transthoracic epicardial pacing for CRT.

**Figure 1:**
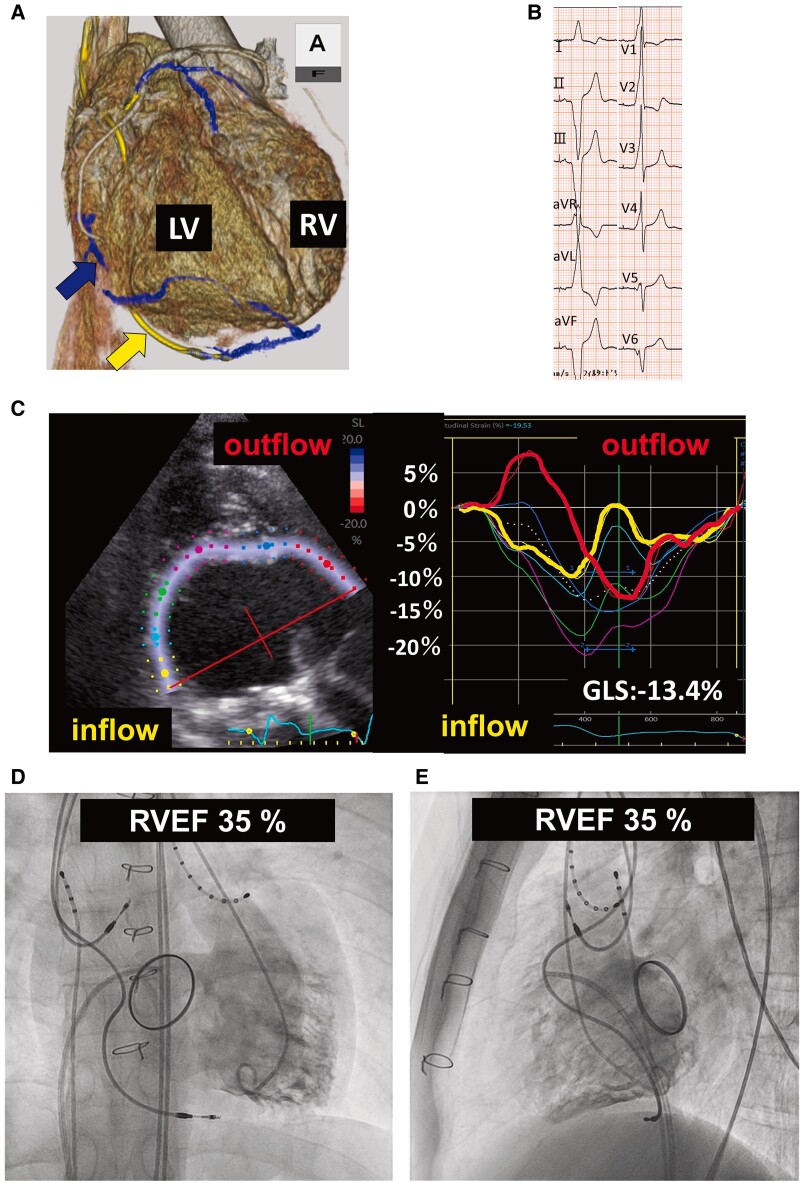
Examinations after right ventricular inflow pacing. *(A)* Computed tomography: the right ventricular lead was coincidently implanted under the dorsal site of the right ventricular inflow via the coronary sinus. The blue and yellow arrows represent the coronary vein and the transvenous lead, respectively. *(B)* Electrocardiogram; QRS duration 188 m. *(C)* Right ventricular speckle tracking echocardiography; the RV outflow was the most delayed contraction site. *(D* and *E)* Right ventricular angiography: right ventricular intraventricular dyssynchrony was evident, and the total right ventricular ejection fraction was 35%. RVEF, right ventricular ejection fraction; GLS, global longitudinal strain.

In the first step, the transvenous atrial (Boston Scientific, Model 7841, IS-1) and ventricular (Boston Scientific, Model 7842, IS-1) leads were implanted. The ventricular lead was unexpectedly placed in the cardiac vein at the dRVI via the proximal coronary sinus (*Figure 1A*). The paced AV interval was set to 180 ms, which was the same as the PR interval before the onset of the complete AV block.

After right atrium and dRVI pacing, the QRS duration was 188 ms (*Figure 1B*). Two months after pacemaker implantation, speckle tracking echocardiography revealed intraventricular dyssynchrony in the RV and delayed contraction in the outflow tract (*Figure 1C*). Echocardiography revealed a RVDd of 45.1 mm and RVDs of 36.6 mm, with an RVEF of 46.6%. Additionally, RVG revealed a RVEDVI of 106 mL/m^2^ and RVEF of 35.0% (*Figure 1D* and *E*).

**Figure 2:**
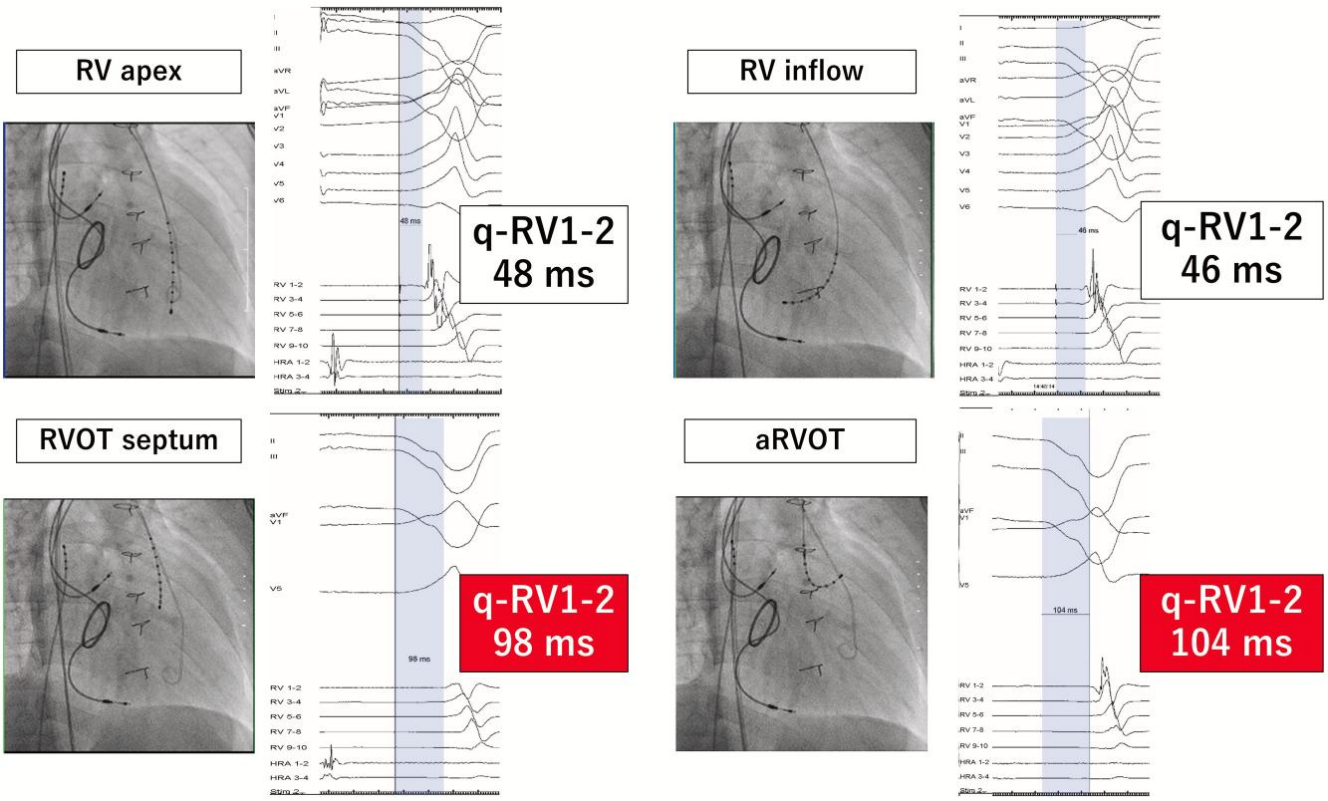
Measurement of q-RV interval at four positions. The q-RV interval was defined as the time from the right ventricular inflow pacing spike to the beginning of the right ventricular electrogram. The anterior free wall was the most delayed conduction position.

**Figure 3:**
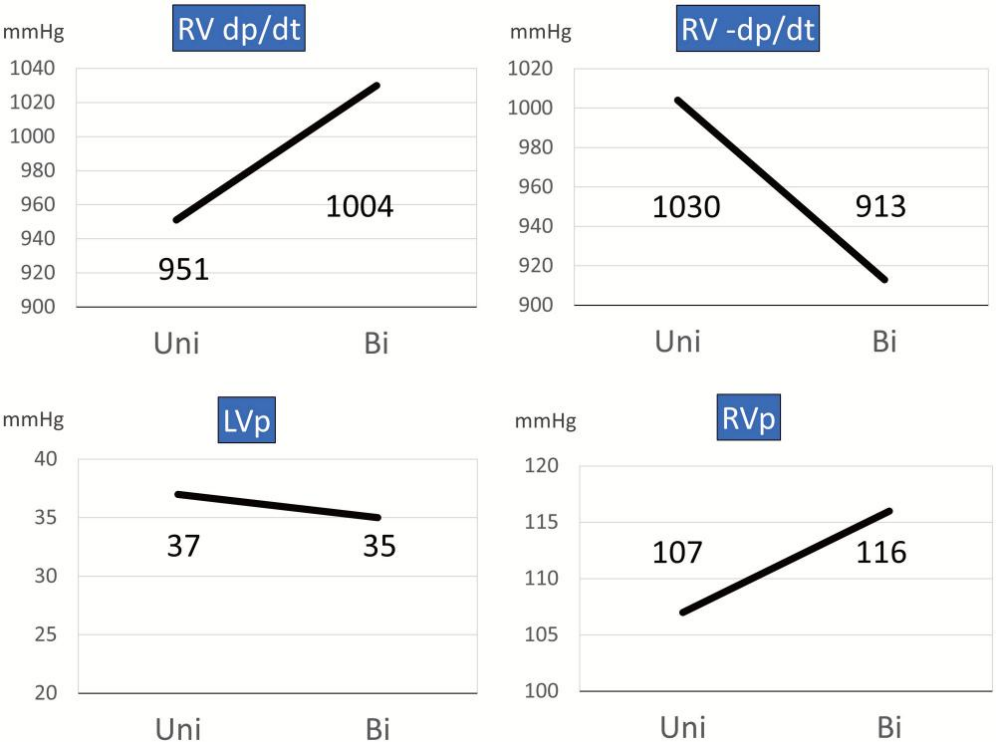
Comparison of pressure study at univentricular pacing and biventricular pacing. RV, right ventricle; LV, left ventricle; RVP, right ventricular pressure; LVP, left ventricular pressure; Uni, univentricular (dorsal right ventricular inflow) pacing; Bi, biventricular (dorsal right ventricular inflow and anterior right ventricular outflow tract) pacing.

For the second step, an electrophysiological examination was performed to evaluate electrical dyssynchrony and the effect of CRT. During dRVI pacing, mapping was performed within the RV and the q-RV interval was measured. An RV electrode was placed at four sites: RV septal apex, RV inflow, RVOT septum, and aRVOT. The conduction time from dRVI to each location was measured to determine where the conduction time was delayed. The results revealed that the aRVOT was the most electrically delayed site (*Figure 2*). Particularly, simultaneous pacing with the dRVI and aRVOT shortened the QRS duration to 132 ms. The RV systolic pressure increased from 107 to 116 mmHg, and RV dp/dt increased from 951 to 1004 mmHg/s (*Figure 3*).

**Figure 4:**
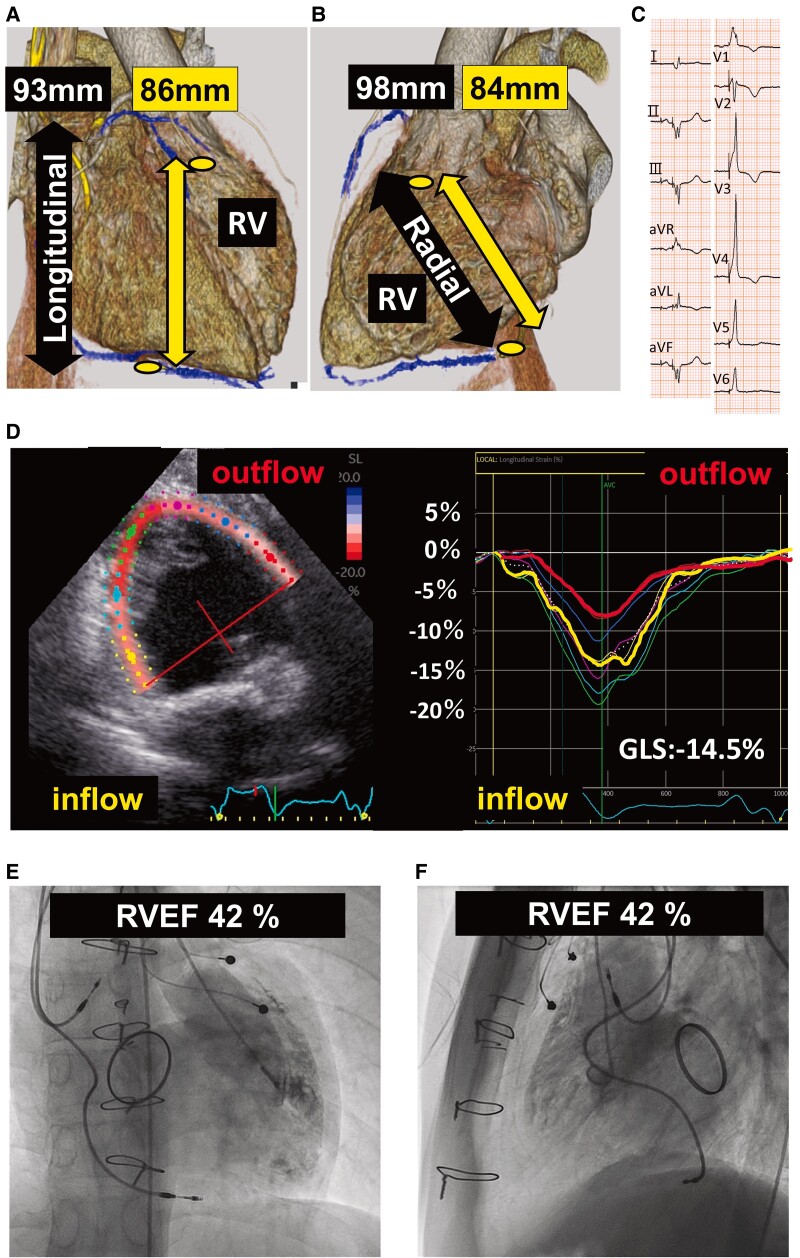
Examinations after biventricular pacing. *(A* and *B)* Computed tomography; right ventricular lead interval was 86 and 84 mm in the longitudinal and radial direction, respectively. It corresponds to 93% and 85% of longitudinal RV length. The two spots represent lead-tip implantation locations. The lower one is the transvenous lead tip, and the upper one is the epicardial lead tip. (*C*) Electrocardiogram; QRS duration, 132 ms. (*D*) Right ventricular angiography: right ventricular intraventricular dyssynchrony improved, and the total right ventricular ejection fraction was 42%. *(E* and *F)* Right ventricular ejection fraction speckle tracking echocardiography showing the disappearance of the right ventricular outflow contraction delay. RVEF, right ventricular ejection fraction; GLS, global longitudinal strain.

For the final step, 5 months after first conventional pacemaker implantation, an epicardial lead was surgically implanted in the aRVOT and placed at the site furthest from the dRVI lead in the longitudinal and radial directions. The inter-lead distance covered 93% (inter-lead distance 86 mm, distance of longitudinal RV length 93 mm) of the total heart in the longitudinal direction and 85% (inter-lead distance 83.5 mm, distance of anteroposterior RV length 97.8 mm) in the radial direction (*Figure 4A* and *B*). The threshold for the aRVOT lead was 2.0 V/0.4 ms.

We programmed AV and VV intervals by evaluating the QRS duration, blood pressure, and central venous pressure during the perioperative period of the CRT upgrade. The paced AV, sensed AV, and VV intervals were set to 180, 130, and 0 ms, respectively.

After simultaneous dRVI and aRVOT pacing, the QRS duration on the ECG decreased to 132 ms and speckle tracking echocardiography indicated that the contractile delay in the RVOT disappeared (*Figure 4C* and *D*). Echocardiography revealed a RVDd of 49.6 mm and RVDs of 37.5 mm, with an RVEF of 56.9%. Right ventriculography displayed an RVEDVI of 106 mL/m^2^ and RVEF of 42.0% (*Figure 4E* and *F*).

Additionally, we re-programmed the AV and VV intervals 8 months after the CRT-P upgrade by evaluating RV dp/dt in the catheter laboratory. We adjusted the AV intervals, such that the paced AV interval was 170 ms and the sensed AV interval was 120 ms. The VV interval remained unchanged at 0 ms.

## Discussion

There are a few reports of CRT using the RV body, RVOT, left ventricular (LV) septal, and RV-free wall with lead implantation.^3^ However, there has been no report of dRVI and aRVOT, as performed in this case. The effectiveness of CRT for ccTGA has been reported; however, the optimal pacing lead position of CRT for ccTGA remains controversial.

The anatomy and contraction characteristics of RV must be understood to consider the optimal lead position of CRT for ccTGA. The subendocardial layer of RV is composed of preferentially arranged longitudinal myocytes from RV apex to papillary muscles and outflow tract. Hence, the predominance of longitudinal subendocardial myocytes and longitudinal shortening accounts for approximately 75% of RV contraction.^4^ However, 3D echocardiography indicates that systemic RV contraction is reduced compared with normal RV.^5^ In the systemic RV, it has been reported that the myocardium of the RV hypertrophies radially.^6^ Additionally, longitudinal strain decreases while radial strain increases. This change is similar to the normal contraction of the LV. The optimal pacing site of the CRT for ccTGA should be considered to complement the longitudinal and radial shortening.

The CRT of a systemic RV with or without a rudimentary LV should be performed with the lead implanted at the furthest sites in the longitudinal direction.^7^ The advantages of this lead position are as follows: the dRVI lead is placed more caudally than the LV apex lead, which makes the anterior aRVOT lead and dRVI lead farther apart in the longitudinal and radial direction. Thus, the longitudinal and radial lead positions still account for 93% and 85% of the RV, respectively. The activation sequence from the dRVI pacing propagates to the RV anterior free wall and subsequently the RV outflow. The dRVI and aRVOT pacing can contract the RV in the longitudinal and radial directions in the shortest time.

We performed a cardiac catheterization examination and identified the latest electrical activation sites, but there are other, less-invasive evaluative methods available. By combining magnetic resonance imaging (MRI) and ECG, it is possible to conduct a more detailed assessment than is allowed by catheterization.^8^ Given the complex structure of congenital heart disease, it is essential to incorporate such evaluation methods moving forward.

We performed CRT upgrade 5 months after pacemaker implantation. When LV pacing is performed for AV block in ccTGA, dyssynchrony appears later.^9^ The electrophysiological study was performed after confirming the appearance of RV dyssynchrony; subsequently, the epicardial lead was implanted in the present case as well.

Initially, we intended to insert a lead into the LV using a fixation lead. However, due to an accidental placement, the lead was instead positioned in the coronary vein. Consequently, the risk of obstruction or damage to the coronary vein was higher, compared with the risk involved if a lead was designed for intravascular use. Of course, the advantage of having pacing from the dRVI at this position is noteworthy, but since this placement deviates from the standard lead insertion methods, the durability of the lead is uncertain. It is necessary to conduct close monitoring.

In this case, RVEF after CRT mildly increased more than before CRT. However, it is lower than before CAVB. This CRT was not intended to improve RV contraction, but to prevent future RV dysfunction caused by single pacing. Pacing the RV in the dorsal region long-term can lead to remodelling due to dyssynchrony. Early CRT was performed before a decrease in EF occurred due to remodelling, which may explain the limited improvement in EF. Other reports have shown little increase in RVEF after CRT.^10^ The evaluation was conducted using RVG and echocardiography. Incorporating MRI evaluations would have allowed for a more detailed assessment.

## Supplementary Material

ytae607_Supplementary_Data

## Data Availability

The data that support the findings of this case report are available from the corresponding author, upon reasonable request.

## Lead author biography

Shigehito Baba graduated from Kanazawa University School of Medicine and currently works as a paediatric cardiologist. He has interest in electrophysiology and cardiac function.

## Notes

**Consent:** We have obtained the patient’s consent to submit the progress of this treatment as a case report. This was recorded in the patient’s medical record on 31 January 2022. This has been agreed with the editors.

**Funding:** None declared.
